# Hospitalization costs of coronaviruses diseases in upper-middle-income countries: A systematic review

**DOI:** 10.1371/journal.pone.0265003

**Published:** 2022-03-11

**Authors:** César Ramos Rocha-Filho, Johnny Wallef Leite Martins, Rosa Camila Lucchetta, Gabriel Sodré Ramalho, Giulia Fernandes Moça Trevisani, Aline Pereira da Rocha, Ana Carolina Pereira Nunes Pinto, Felipe Sebastião de Assis Reis, Laura Jantsch Ferla, Patrícia de Carvalho Mastroianni, Luci Correa, Humberto Saconato, Virgínia Fernandes Moça Trevisani

**Affiliations:** 1 Evidence-Based Health Program, Escola Paulista de Medicina, Universidade Federal de São Paulo, São Paulo, SP, Brazil; 2 Pharmaceutical Sciences Program, School of Pharmaceutical Sciences, Universidade Estadual de São Paulo, Araraquara, SP, Brazil; 3 Department of Drugs and Medicines, School of Pharmaceutical Sciences, Universidade Estadual de São Paulo, Araraquara, SP, Brazil; 4 Department of Sustainability and Social Responsibility, Hospital Alemão Oswaldo Cruz, São Paulo, SP, Brazil; 5 Escola Paulista de Medicina, Universidade Federal de São Paulo, São Paulo, SP, Brazil; 6 Faculdade de Medicina, Universidade de Santo Amaro, São Paulo, SP, Brazil; 7 Department of Biological and Health Sciences, Universidade Federal do Amapá, Macapá, AP, Brazil; 8 Department of Medical Practices, Beneficência Portuguesa de São Paulo, São Paulo, SP, Brazil; 9 Discipline of Infectiology, Escola Paulista de Medicina, Universidade Federal de São Paulo, São Paulo, SP, Brazil; 10 Discipline of Emergency and Evidence-Based Medicine, Escola Paulista de Medicina, Universidade Federal de São Paulo, São Paulo, SP, Brazil; Shahid Beheshti University of Medical Sciences, ISLAMIC REPUBLIC OF IRAN

## Abstract

**Background:**

COVID-19, SARS and MERS are diseases that present an important health burden worldwide. This situation demands resource allocation to the healthcare system, affecting especially middle- and low-income countries. Thus, identifying the main cost drivers is relevant to optimize patient care and resource allocation.

**Objective:**

To systematically identify and summarize the current status of knowledge on direct medical hospitalization costs of SARS, MERS, or COVID-19 in Upper-Middle-Income Countries.

**Methods:**

We conducted a systematic review across seven key databases (PubMed, EMBASE, BVS Portal, CINAHL, CRD library, MedRxiv and Research Square) from database inception to February 2021. Costs extracted were converted into 2021 International Dollars using the Purchasing Power Parity-adjusted. The assessment of quality was based on the protocol by the BMJ and CHEERS. PROSPERO 2020: CRD42020225757.

**Results:**

No eligible study about SARS or MERS was recovered. For COVID-19, five studies presented cost analysis performed in Brazil, China, Iran, and Turkey. Regarding total direct medical costs, the lowest cost per patient at ward was observed in Turkey ($900.08), while the highest in Brazil ($5,093.38). At ICU, the lowest was in Turkey ($2,984.78), while the highest was in China ($52,432.87). Service care was the most expressive (58% to 88%) cost driver of COVID-19 patients at ward. At ICU, there was no consensus between service care (54% to 87%) and treatment (72% to 81%) as key burdens of total cost.

**Conclusion:**

Our findings elucidate the importance of COVID-19 on health-economic outcomes. The marked heterogeneity among studies leaded to substantially different results and made challenging the comparison of data to estimate pooled results for single countries or regions. Further studies concerning cost estimates from standardized analysis may provide clearer data for a more substantial analysis. This may help care providers and policy makers to organize care for patients in the most efficient way.

## Introduction

Within the first two decades, the 21st century was marked by three coronavirus outbreaks: Severe Acute Respiratory Syndrome (SARS) Coronavirus (SARS-CoV), Middle East Respiratory Syndrome (MERS) Coronavirus (MERS-CoV) and, most recently, the SARS Coronavirus 2 (SARS-CoV-2) [1].

The SARS-CoV epidemic occurred from 2002 to 2003, presenting no new cases since 2004, totalizing 8,422 cases and 916 deaths, with a fatality rate of 11% [2]. MERS-CoV, from 2012 to 2018, infected over 2,000 people globally, mostly in Middle Eastearn countries. In Saudi Arabia, for example, the fatality rate in this period was 35.67% [3]. SARS-CoV-2 emerged by the end of 2019 and, as of first semester of 2021, there have been over 200 million confirmed cases and over 4 million deaths worldwide [4].

Along with the marked clinical impact, the battle against the coronaviruses demands several resources, elevating the healthcare costs. Healthcare systems and national governments may use data on the economic burden of infectious diseases as evidence to make informed decisions to allocate limited resources optimally and to prioritize interventions in their public or local policies [5,6].

In middle- and low-income economies, specifically, the protection of populations from financial difficulties has been a goal of global health policy development [5]. In these countries, the high economic burden is often combined with limited access to health care facilities and low quality of services. Therefore, a growing need for understanding the diseases economic consequences is commonly reported [6].

Given the high prevalence of infection due to coronaviruses, it is essential to illuminate potentially cost-saving or main cost drives to inform resource allocation decisions and affordability of interventions. Thus, the objective of this study was to identify and assess the current evidence about the costs of managing hospitalized patients diagnosed with SARS, MERS, or Coronavirus Disease 2019 (COVID-19) in Upper-Middle-Income Countries (UMIC) through a Systematic Review. Our goal was to compare cost estimates between countries with a similar economic development level and examine relevant cost drivers.

## Methods

### Study design and registration

We performed a SR following the recommendations proposed by the Cochrane Reviews network [7], adapting the processes of quality assessment and data analysis [8]. This is the first publication of an umbrella SR of economic burden of viral Acute Respiratory Infections (ARI) in UMCI, registered at the International Prospective Register of Systematic Reviews (PROSPERO) [identifier: CRD42020225757] with a protocol published prior to starting the literature search [9]. The Preferred Reporting Items for Systematic Reviews and Meta-Analyses (PRISMA) guidelines was used as a basis for the overall study approach [10].

### Search strategy

The search strategy was developed by a specialist. PubMed, EMBASE, BVS Portal, CINAHL, CRD library, and the preprint platforms MedRxiv and Research Square, were searched on 15 February 2021. Relevant literature was identified by using three categories of keywords: (1) viral ARI, (2) cost analysis, and (3) developing countries. The terms were translated into the query language of each database. No filter of year of publication or language was applied [9]. See supplementary file for database queries and resulting yields (S1 Table).

If a review study on the topic was retrieved from the search, we scooped the reference list to collect further eligible studies. The same process was performed with the selected manuscripts. Contact with article authors was made to capture full-text article of recovered abstracts that potentially could be included in our SR [9].

### Study selection

We uploaded the records identified through database search to the web-based bibliographic manager Rayyan QCRI [11]. After removing duplicates, two review authors (CRRF and GSR) independently screened the titles and abstracts of the records. An article was considered for inclusion in case it discussed individually the direct medical costs for hospitalization due to COVID-19, MERS, or SARS in an UMIC, stablished according to the World Bank classification (2019 GNI) [12].

Our selection was limited to human subjects and studies that estimated costs based on primary partial economic evaluation, such as cost-of-illness (COI) studies, cost analysis, observational reports (cross-sectional studies, and prospective and retrospective cohort), or economic modelling studies. Letters, reviews, commentaries, editorials, case reports, case series, and papers without sufficient information to clearly identify methods, sources, or unit costs were excluded [9].

To determine the final eligibility, the two investigators independently assessed the full text of records screened. Disagreements were resolved by the involvement of a third investigator (HS or LC). Abstracts that could not be recovered full-text and non-Roman language reports from which was not possible to perform a valid translation (e.g., Arabic, Chinese, and Russian) were included into the review and filed as “Studies Awaiting Classification” [13].

### Data extraction

Data were extracted by one reviewer (CRRF), according to a standardized data extraction form previously pre-tested on a sample of 3 studies. All extracted data were double checked by a second reviewer (JWLM), and any differences in point of view were discussed with a third researcher (HS or RCL). Costs extracted were converted into 2021 International Dollars (Int$ 2021) using the Purchasing Power Parity (PPP)-adjusted proposed on the cost converter tool from CCEMG-EPPI Centre (https://eppi.ioe.ac.uk/costconversion/) [14]. This method was applied to achieve better comparability between different currencies [5,6,8].

### Study assessment

Criteria to assess methodological strengths and weaknesses in study design were adapted from a previous costing review by Oliveira, Itria, and Lima [8], that was based on the protocol proposed by the British Medical Journal (BMJ) [15], and on the Consolidated Health Economic Evaluation Reporting Standards (CHEERS) [16]. All included studies were independently appraised by two reviewers (CRRF and JWLM). The results were confronted, and any discrepancies discussed with a third reviewer (RCL).

### Data synthesis

The data extracted was stratified, synthesized, and narratively reported per inpatient care in Intensive Care Units (ICU) or routine care on the wards. Based on previously SR of cost analyses [6,8], we stablished four cost categories to identify dominant cost drivers: treatment, diagnostic tests, hospital bed/day or routine service costs, and others. When only total cost per variable was presented, we calculated the individual value based on population sample. The quality assessment analysis was plotted using the tool provided by McGuinness and Higgins [17] (https://mcguinlu.shinyapps.io/robvis/).

## Results

### Literature search

**Fig 1:**
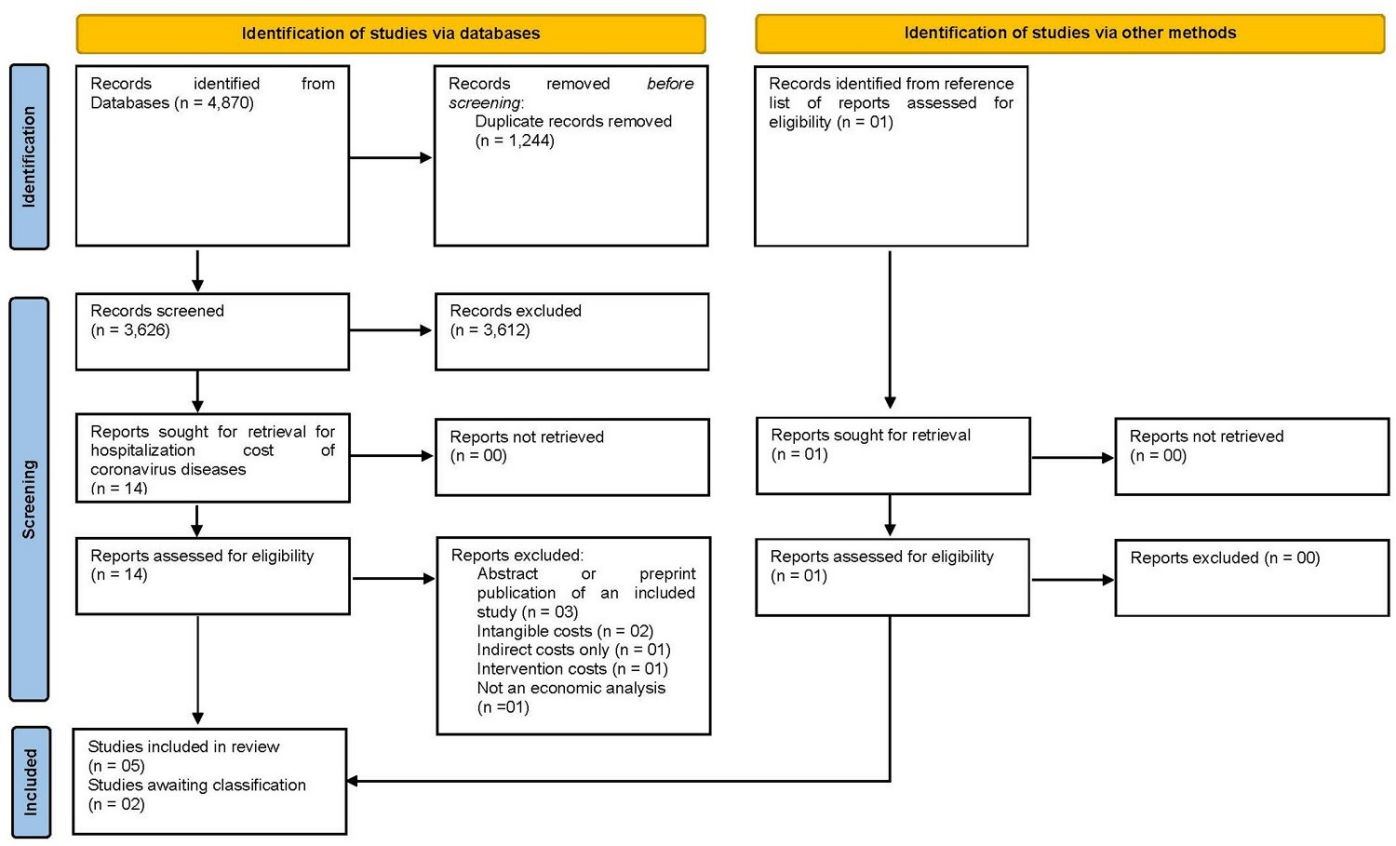
PRISMA flow diagram. *Adapted from Page et al*. *(2021)* [10].

The initial databases search yielded 4,870 references to systematically assess. An additional study identified through manual research was also eligible. After duplicate studies were removed, the titles and abstracts from 3,626 records were screened for inclusion, of which 14 articles corresponded to hospitalization costs of SARS, MERS, or COVID-19. By the end, five unique studies met our eligibility criteria and were included in the review. There were two additional studies “awaiting classification”. One we were unable to translate [18] and the other we were unable to obtain the full text [19], even after contacting study authors. See the PRISMA Flow Diagram [10] of the study selection in Fig 1 and the characteristics of excluded studies and studies awaiting classification in S2 and S3 Tables in supporting information.

### Characteristics of the studies

All five studies appraised in this SR discussed the COVID-19 direct medical hospitalization costs. The main characteristics are summarized in Table 1. The only manuscript recovered that discussed SARS costs was labeled in the “studies awaiting classification”. Our search strategy did not identify any eligible study on MERS hospitalization costs.

**Table 1:** Characteristics of included studies (COVID-19 hospitalization costs).

*Study ID*	*Setting*, *year of costing*	*Study design*	*Perspective*	*Resource Quantification*	*Epidemiological Approach*
Darab et al. (2021) [20]	Iran, 2020	CS	Household	Bottom-up	Incidence
Gedik (2020) [21]	Turkey, 2020	CA	Healthcare system	Not reported	Not reported
Jin et al. (2021) [22]	China, 2019	COI	Healthcare system	Bottom-up	Not reported
Liang et al. (2020) [23]	China, 2020	CA	Healthcare system	Bottom-up	Not reported
Miethke-Morais et al. (2020) [24]	Brazil, 2020	CS	Health facility	Bottom-up	Not reported

Abbreviations: CA—Cost Analyses; CS—Cross-Sectional; COI—Cost-of-Illness.

### Hospitalization cost of COVID-19

The inpatient care costs of COVID-19 at ward and ICU are detailed in Tables 2 and 3, respectively. Gedik did not show the separated costs, presenting only the total value [21]. Liang et al. was the only study that showed the median data [23]. Based on the definitions presented by Jin et al., we accepted non-severe cases as inpatient care at ward, and severe and critical cases as inpatient care at ICU [22].

**Table 2:** Direct medical costs of inpatients with COVID-19 at ward. Costs were adjusted into Int$2021.

*Study ID*	*Hospitalization days*	*Treatment*	*Diagnostic tests*		*Hospital bed/day or Routine Service costs*	*Others*
**Darab et al. (2020)** [20]	Not reported	Drugs and supplies: $802.34 Rehabilitation and Dialysis: $23.48	Electrography and lab: $306.24 Imaging: $88.81		Bed/stay: $1,195.34 Physician visit: $455.27 Nursing: $65.33 Surgeon: $61.25	Other services ^b^: $40.83
**Total direct medical costs**				**$3,040.92**		
**Gedik (2020)** [21]	8.97 days	Not reported	Not reported		Not reported	Not reported
**Total direct medical costs**				**$900.08**		
**Jin et al. (2021)** [22]	14 days	Drugs: $114.23 TPEC: $11.34	Identification and diagnosis ^c^: $84.95 Identification and diagnosis ^d^: $89.73		Inpatient care: $1,617.28	Follow-up: $7.48
**Total direct medical costs**				**$1,925.00**		
**Liang et al. (2020)** ^**a**^ [23]	20 days	Drugs: $425.29 Supplies: $542.97 Treatment: $298.27	Lab. tests: $909.10		Bed/stay: $525.23 Medical examination: $246.88 Nursing: $69.38 Consultation: $60.10	Others ^b^: $39.36
**Total direct medical costs**				**$3,510.48**		
**Miethke-Morais et al. (2020)** [24]	Not reported	Drugs: $141.60 Supplies: $124.87 Blood components: $51.93	Lab. tests: $93.96 Rad. Exam: $36.08		Nonmedical staff: $2,970.66 Medical staff: $1,185.84 DFC: $330.46	PPE: $154.31 Nutrition: $3.66
**Total direct medical costs**				**$5,093.38**		

Data are presented as mean per patient, except for Liang et al. (2020) which is ^a^ presented as median per patient. ^b^ See supplementary file. ^c^ Identified from close contacts. ^d^ Identified from suspected cases. *Abbreviations*: DFC—Daily Fixed Costs; Lab—Laboratory; PPE—Personal Protective Equipment; Rad—Radiologic; TPEC—Treatment for pre-existing conditions.

**Table 3:** Direct medical costs of inpatients with COVID-19 at ICU. Costs were adjusted into Int$2021.

*Study ID*	*Hospitalization days*	*Treatment*	*Diagnostic tests*		*Hospital bed/day or Routine Service costs*	*Others*
**Darab et al. (2020)** [20]	11 days	Drugs and supplies: $4,635.39 Rehabilitation and Dialysis: $349.11	Electrography and lab: $1,007.52 Imaging: $181.70		Bed/stay: $6,209.00 Physician visit: $574.70 Nursing: $270.51 Surgeon: $233.76	Other services ^b^: $81.66
**Total direct medical costs**				**$13,542.77**		
**Gedik (2020)** [21]	14.74 days	Not reported	Not reported		Not reported	Not reported
**Total direct medical costs**				**$2,984.78**		
**Jin et al. (2021)** ^**e**^ [22]	28 days	Drugs: $11,587.13 Oxygen therapy: $113.73 TPEC: $1,393.54	Identification and diagnosis ^c^: $84.95 Identification and diagnosis ^d^: $89.73		Inpatient care: $4,923.99	Follow-up: $7.48
**Total direct medical costs**				**$18,200.55**		
**Jin et al. (2021)** ^**f**^ [22]	42 days	Drugs: $20,289.83 Tracheal intubation: $51.92 TPEC: $1,558.30 MV: $8,964.64 ECMO: $6,229.86 Artificial Kidney: $4,306.91 Plasma exchange: $1,235.59	Identification and diagnosis ^c^: $84.95 Identification and diagnosis ^d^: $89.73		Inpatient care: $9,613.69	Follow-up: $7.48
**Total direct medical costs**				**$52,432.87**		
**Liang et al. (2020)**^**a**^ [23]	27 days	Drugs: $1,668.49 Supplies: $1,544.09 Treatment: $2,053.87	Lab. tests: $2,990.94		Bed/stay: $1,103.00 Medical examination: $476.86 Nursing: $87.58 Consultation: $81.57	Not reported
**Total direct medical costs**				**$11,345.92**		
**Miethke-Morais et al. (2020)** [24]	Not reported	Drugs: $660.83 Supplies: $502.92 Blood components: $212.50	Lab. tests: $370.51 Rad. Exam: $38.65		Nonmedical staff: $7,929.14 Medical staff: $5,059.85 DFC: $1,136.20	PPE: $329.85 Nutrition: $44.74
**Total direct medical costs**				**$16,285.19**		

Data are presented as mean per patient, except for Liang et al. (2020) which is ^a^ presented as median per patient. ^b^ See supplementary file. ^c^ Identified from close contacts. ^d^ Identified from suspected cases. ^e^ Severe case. ^f^ Critical case. *Abbreviations*: DFC—Daily Fixed Costs; ECMO—Extracorporeal Membrane Oxygenation; Lab—Laboratory; MV—Mechanical Ventilation; PPE—Personal Protective Equipment; Rad—Radiologic; TPEC—Treatment for pre-existing conditions.

### Cost drivers

**Fig 2:**
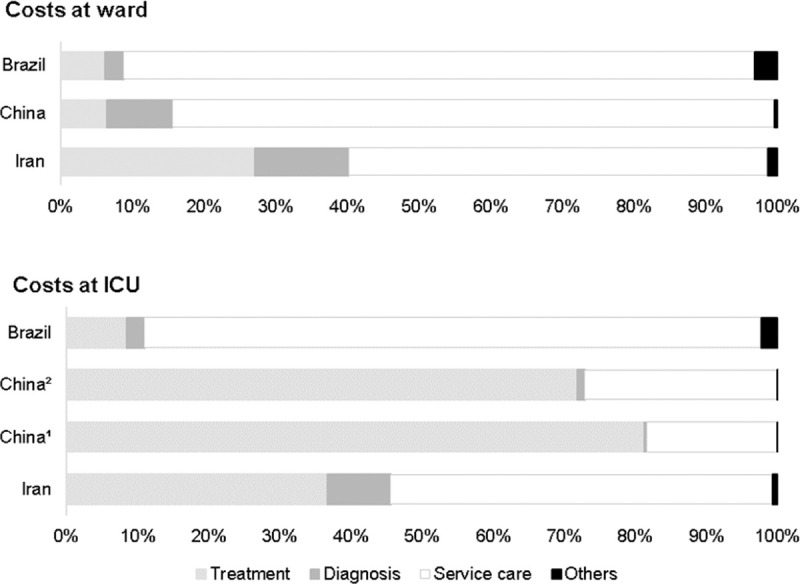
Cost drivers of hospitalization due to COVID-19 at ward and ICU per country. Data are presented as a percentage (%) of total cost. ^1^Severe case. ^2^Critical case.

Fig 2 presents the percentage of consumption of total cost for the four cost categories: treatment, diagnosis, service care, and others. The studies that did not report any cost category [21] or presented data as median [23] were not included in this graphic.

At ward, the findings among included studies were similar, reporting hospital bed/day or routine service costs (service care) as the most dominant cost driver, ranging between 58% to 88% of total cost. The same homogeneity was not observed among data at ICU. Two studies remained service care as dominant cost drivers (54% to 87%) [20,24], while Jin et al. appointed treatment category consuming 72% to 81% of total cost [22].

### Report assessment of the included studies

**Fig 3:**
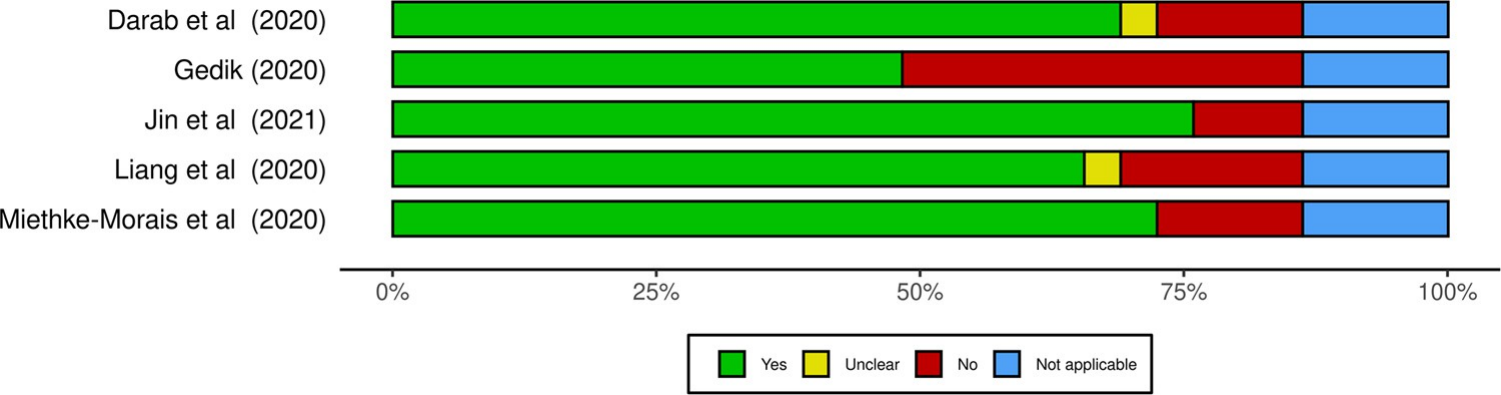
Methodology assessment of included studies based on the checklist provided by Oliveira, Itria and Lima [8]. Graphic produced with *robvis*, provided by McGuinnes and Higgins [17].

Fig 3 presents a summary plot of the methodology assessment of included studies. Overall, most of the studies (4/5) satisfactorily reported their methodology and results (more than 60% of “yes” answers) [8]. The questions with more “no” answers were related to the statement of epidemiological approach (Table 1), characterization of uncertainty and heterogeneity, and report of sensitivity analysis. Supplementary file presents a traffic-light plot with detailed assessment per question (S1 Fig).

## Discussion

This SR synthetizes the available evidence relating the direct costs of hospitalization due to coronavirus diseases from countries with similar economic development levels. Of the three infections investigated, no eligible study about the economic impact of SARS and MERS was recovered. For COVID-19, financial burden was presented in five studies in Brazil, China, Iran, and Turkey.

Comparing the total direct medical costs, the lowest cost per patient at ward was observed in Turkey ($900.08), while the highest in Brazil ($5,093.38) (Int$2021). At ICU, the lowest cost once again was reported by the Turkish study ($2,984.78), while the highest was attributable to a study developed in China ($52,432.87). Analyzing the cost drivers of COVID-19 patients at ward, the most dominant was attributable to service care (58% to 88%). For patients at ICU, there was no consensus among studies that reported either service care (54% to 87%) or treatment (72% to 81%) as key burdens of total cost.

The magnitude observed on the cost drivers and estimates highlights a marked heterogeneity among evidence, which prevents us from drawing firm conclusions on the current cost burden. Thus, any generalizability of the results to a regional or global level should be taken with caution.

The disparities in the reported costs between articles included in our review can be partially explained by the lack of standardization of costing methods, exposed in Tables 1–3. Although among studies there is an individual report quality (Fig 3), we observed that important components of case management costs were not consistently presented. This finding is commonly reported in SRs of economic analysis, which makes comparison of data or metanalysis more challenging [6,8,25].

Overall, the studies poorly described their individuals. For example, only Gedik reported the mean age of the sample [21], while only Miethke-Morais et al. highlighted patients’ comorbidities as an increase factor of the total cost of COVID-19 [24]. It is important to observe that all included studies were developed between March and August 2020, when the knowledge of the disease was expanding. Nowadays, it is known that these factors, among others, strongly contribute to poor prognosis of COVID-19 [26] and, as a result, probable predictors of high use of health resources and costs.

In this scenario, overall, patients need more care consumables, as specific treatment, long hospital and critical care stays, which potentially increase costs [27]. In fact, Jin et al. reported treatment as the most dominant cost driver for severe and critical patients (72% and 81%, respectively) [22]. Thus, we strongly recommend that future cost studies on COVID-19 consider and analyze baseline risk status.

Access to technologies seems to be another solid hypothesis to explain the heterogeneity among reported costs. Although the studies were from a similar economic level, gaps and peculiarity in local health systems may hamper the access to diagnosis, supplies, and treatments. Generally, due to different levels of taxation, transportation cost, procurement practices, intellectual property and patents, different prices for the same technology are expected across borders [28].

This inequality is not restricted to COVID-19 pandemic. In fact, studies have shown that differences in access to early diagnosis and optimum treatment are directly related to survival trends and costs of different illnesses, such as cancer [29]. Based on our findings, it is not clear how much this variable impacted heterogeneity. However, analyzing the technologies reported, Jin et al. incorporated a health surveillance process and advanced treatments that were not presented by other authors in their cost analysis, which may explain the high cost per patient [22].

Another important aspect that we should consider about consistency across studies is the strength of evidence. Although the tool used for report assessment of included studies does not translate to quality of evidence, it gives us some observations [8].

For both analyses, at ward and ICU, findings from Gedik were the one that most diverged from the other economic evidences [21]. In the same manner, the Turkish study was the only one with an unsatisfactory report (less than 50% of “yes” answers). This low index of the report may be understood as a weak cost analysis.

Apart from the methodological and contextual differences between studies, it appears that service care is a dominant cost driver for COVID-19 hospitalization cost. These findings can be explained based on the current knowledge of patient management. To date, there is no standardized drug therapy for the treatment of COVID-19, varying across health institutions. Patient care must follow monitoring and symptom management, which may consume medical labor [27].

### Strengths and limitations

To the best of our knowledge, this is the first SR specifically focusing on coronavirus hospitalization costs in UMIC. Results can be relevant to support future cost analyses and public policy. However, certain limitations must be acknowledged.

First, we assumed a potential publication bias due to the coronavirus topic. As known, the outbreaks of SARS, MERS and COVID-19 started in Asiatic countries. Thus, an important number of scientific productions may be developed and published in specific journals and languages, not always indexed in the great databases, or recovered by our search strategy. In fact, our search strategy could not recover studies on SARS or MERS. However, for COVID-19, this limitation does not appear to be significant. Due to the striking heterogeneity of cost analysis, we believe that more studies could not add new information.

Second limitation that should be reported is that we did not include full cost analysis, such as cost-benefit and cost-effectiveness. The implementation of this exclusion criteria was based on the previous SR of economic burden. The reports discussed that although this study design may present primary cost evaluation, the proportion of studies that effectively are not based on secondary sources does not impact in the review results [30]. That said, the importance of this limitation for the overall interpretation of our SR is expected to be minor.

Another limitation refers to the date of the search (February 2021) which may have been responsible for identifying studies that covered only the first wave of COVID-19. Despite of that, this review is expected to have its value as it presents a complete analysis of this period of the pandemic and can be compared with future analyzes referring to more recent periods.

## Conclusion

In conclusion, our review elucidates the importance of COVID-19 on health-economic outcomes. Due to a considerable heterogeneity across costing methodologies, we observed a great variation in terms of absolute costs per patient, making comparison challenging even among countries with similar economic status. However, the appraised studies show that direct medical costs may encompass multiple dimensions. Cost drivers were mainly service care and treatment. As expected, total costs increased with greater disease severity.

Although not transferable to other settings, these data should help readers to understand and contextualize the magnitude of COVID-19 economic burden. The comprehension about the cost drivers as well as the total cost is a key step to informing national priority-setting around COVID-19 prevention and control interventions. Obtaining cost estimates from standardized analysis will provide a clearer perspective and comparative assessment to act.

## Supporting information

S1 Checklist PRISMA checklist. (DOC)

S1 Fig “Traffic-light” plots of the domain-level judgements for each individual result. (TIF)

S1 Table Search strategy and recovered results. (DOC)

S2 Table Characteristics of excluded studies. (DOC)

S3 Table Characteristics of studies awaiting classification. (DOC)

S4 Table Medical cost categories definitions reported in the included studies. (DOC)

S5 Table Report assessment tool. (DOC)

S1 Appendix Supporting information references. (DOC)

S1 Protocol Protocol for systematic review. (DOC)
