# Supplementary figures and images for: Hospitalization costs of coronaviruses diseases in upper-middle-income countries: A systematic review
Source: PLoS One. 2022 Mar 11;17(3):e0265003. doi: 10.1371/journal.pone.0265003 (PMC8916657; doi:10.1371/journal.pone.0265003)

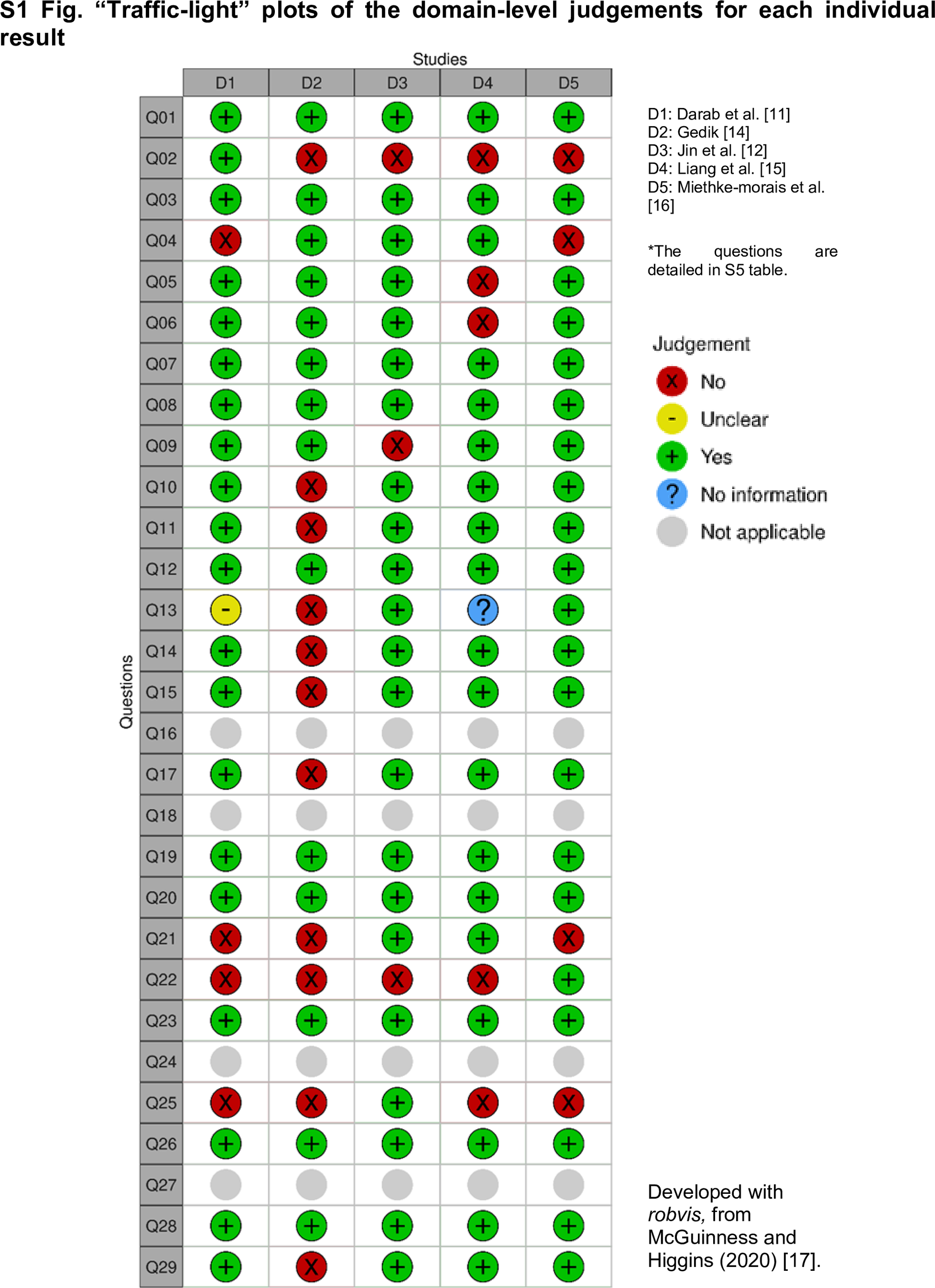

Supplement: S1 Fig — (TIF) [file pone.0265003.s002.tif]
