# Supplementary material for: Hospitalization costs of coronaviruses diseases in upper-middle-income countries: A systematic review
Source: PLoS One. 2022 Mar 11;17(3):e0265003. doi: 10.1371/journal.pone.0265003 (PMC8916657; doi:10.1371/journal.pone.0265003)
Supplement: S1 Table — (DOC) [file pone.0265003.s003.doc]

# S1 Table. Search strategy and recovered results

*Search date: 02-15-2021*

| **Database: Medline via Pubmed** | |
| --- | --- |
| #1 | "Respiratory Tract Infections/economics"[Mesh] OR "Coronavirus Infections/economics"[Mesh] OR "COVID-19/economics"[Mesh] OR "Severe Acute Respiratory Syndrome/economics"[Mesh] OR "Influenza, Human/economics"[Mesh] OR "Respiratory Syncytial Virus Infections"[Majr] OR "Parainfluenza Virus 3, Human"[Majr] OR "Bronchiolitis, Viral/economics"[Mesh] OR "Pneumonia, Viral/economics"[Mesh] OR "Adenovirus Infections, Human/economics"[Mesh] OR "respiratory infection*"[tiab] OR "acute respiratory infection*"[tiab] OR "viral respiratory infection*"[tiab] OR "viral respiratory disease*"[tiab] OR "acute respiratory tract infection*"[tiab] OR "airway infection*"[tiab] OR "pulmonary tract infection"[tiab] OR "coronavirus*"[tiab] OR "SARS-CoV*"[tiab] OR "covid*"[tiab] OR "severe acute respiratory syndrome"[tiab] OR "SARS"[tiab] OR "acute respiratory disease*"[tiab] OR "middle-east respiratory syndrome"[tiab] OR "MERS"[tiab] OR "Syncytial Virus*"[tiab] OR "Viral pneumonia*"[tiab] OR "Parainfluenza Virus Type 3"[tiab] OR "Para influenza virus type 3"[tiab] OR "Para-influenza virus type 3"[tiab] OR "Human parainfluenza virus 3"[tiab] OR "bronchiolitis"[tiab] OR "influenza"[tiab] OR "influenza like illness"[tiab] OR "influenza-like illness"[tiab] OR "influenza like symptom"[tiab] OR "influenza like syndrome"[tiab] OR "flu like illness"[tiab] OR "flu like symptom*"[tiab] |
| #2 | "Developing Countries/economics"[Mesh] OR "middle-income"[tiab] OR "upper-middle-income"[tiab] OR "least developed countries"[tiab] OR "under developed nation*"[tiab] OR "underdeveloped*"[tiab] OR "third-world*"[tiab] OR "thirdworld*"[tiab] OR "3rd-world"[tiab] OR "Albania"[tiab] OR "American Samoa"[tiab] OR "Argentina"[tiab] OR "Armenia"[tiab] OR "Azerbaijan"[tiab] OR "Belarus"[tiab] OR "Belize"[tiab] OR "Bosnia and Herzegovina"[tiab] OR "Botswana"[tiab] OR "Brazil"[tiab] OR "Bulgaria"[tiab] OR "China"[tiab] OR "Colombia"[tiab] OR "Costa Rica"[tiab] OR "Cuba"[tiab] OR "Dominica"[tiab] OR "Dominican Republic"[tiab] OR "Ecuador"[tiab] OR "Equatorial Guinea"[tiab] OR "Fiji"[tiab] OR "Gabon"[tiab] OR "Georgia"[tiab] OR "Grenada"[tiab] OR "Guatemala"[tiab] OR "Guyana"[tiab] OR "Indonesia"[tiab] OR "Iran"[tiab] OR "Islamic*"[tiab] OR "Iraq"[tiab] OR "Jamaica"[tiab] OR "Jordan"[tiab] OR "Kazakhstan"[tiab] OR "Kosovo"[tiab] OR "Lebanon"[tiab] OR "Libya"[tiab] OR "Malaysia"[tiab] OR "Maldives"[tiab] OR "Marshall Islands"[tiab] OR "Micronesia"[tiab] OR "Mexico"[tiab] OR "Montenegro"[tiab] OR "Namibia"[tiab] OR "North Macedonia"[tiab] OR "Paraguay"[tiab] OR "Peru"[tiab] OR "Russian*"[tiab] OR "Samoa"[tiab] OR "Serbia"[tiab] OR "South Africa"[tiab] OR "Saint Lucia"[tiab] OR "Saint Vincent*"[tiab] OR "Suriname"[tiab] OR "Thailand"[tiab] OR "Tonga"[tiab] OR "Turkey"[tiab] OR "Turkmenistan"[tiab] OR "Tuvalu"[tiab] OR "Venezuela*"[tiab] |
| #3 | "Economic*"[tiab] OR "Income*"[tiab] OR "Expenditure*"[tiab] OR "cost-of-illness"[tiab] OR "cost of illness"[tiab] OR "cost of sickness"[tiab] OR "financial burden"[tiab] OR "financial impact"[tiab] OR "economic burden"[tiab] OR "burden of illness"[tiab] OR "out-of-pocket*"[tiab] OR "health expenditure*"[tiab] OR "budget*"[tiab] OR "fee"[tiab] OR "direct cost*"[tiab] OR "cost allocation"[tiab] OR "resource allocation*"[tiab] OR "allocative efficiency"[tiab] OR "payment*"[tiab] OR "cost analysis"[tiab] OR "macroeconomic*"[tiab] OR "microeconomic*"[tiab] OR "pricing"[tiab] OR "Cost measure*"[tiab] OR "Illness Burden*"[tiab] OR "sickness cost*"[tiab] OR "disease cost*"[tiab] OR "Illness cost*"[tiab] OR "direct service cost*"[tiab] OR "service cost*"[tiab] OR "hospital charge*"[tiab] OR "hospital cost*"[tiab] OR "hospitalization cost*"[tiab] OR "medical cost*"[tiab] OR "medical care cost*"[tiab] OR "health care cost*"[tiab] OR "medical burden"[tiab] OR "expense"[tiab] OR "spending"[tiab] OR "Markov*"[tiab] OR "Monte Carlo Method"[tiab] OR "Decision Trees"[tiab] |
| #4 | #1 AND #2 AND #3 |
| **Number of records identified:** 2,437 | |

| **Database: EMBASE via Elsevier** | |
| --- | --- |
| #1 | 'respiratory tract infection'/mj OR 'Coronavirus infection'/mj OR 'severe acute respiratory syndrome'/mj OR 'viral respiratory tract infection'/mj OR 'SARS-related coronavirus'/mj OR 'pandemic influenza'/mj OR 'flu like syndrome'/mj OR 'influenza'/mj OR 'seasonal influenza'/mj OR 'respiratory syncytial virus infection'/mj OR 'Human respiratory syncytial virus'/mj OR 'viral bronchiolitis'/mj OR 'Middle East respiratory syndrome coronavirus'/mj OR 'Human parainfluenza virus 3'/mj OR 'virus pneumonia'/mj OR 'coronavirus disease 2019'/exp OR "respiratory infection*":ab,ti OR "acute respiratory infection*":ab,ti OR "acute respiratory tract infection*":ab,ti OR "airway infection*":ab,ti OR "pulmonary tract infection":ab,ti OR "viral respiratory infection*":ab,ti OR "viral respiratory disease*":ab,ti OR "respiratory disease":ab,ti OR "coronavirus*":ab,ti OR "SARS-CoV*":ab,ti OR "covid*":ab,ti OR "pneumonia":ab,ti OR "severe acute respiratory syndrome":ab,ti OR "SARS":ab,ti OR "acute respiratory disease*":ab,ti OR "middle-east respiratory syndrome":ab,ti OR "MERS":ab,ti OR "Syncytial Virus*":ab,ti OR "Viral pneumonia*":ab,ti OR "Para influenza virus type 3":ab,ti OR "Para-influenza virus type 3":ab,ti OR "Parainfluenza Virus Type 3":ab,ti OR "Human parainfluenza virus 3":ab,ti OR "para influenza 3":ab,ti OR "parainfluenza 3*":ab,ti OR "bronchiolitis":ab,ti OR "influenza":ab,ti OR "influenza like illness":ab,ti OR "influenza-like illness":ab,ti OR "influenza like symptom":ab,ti OR "influenza like syndrome":ab,ti OR "flu like illness":ab,ti OR "flu like symptom*":ab,ti OR "acute influenzal pneumonia":ab,ti |
| #2 | 'developing country'/mj OR 'middle income country'/mj OR "middle-income":ab,ti OR "upper-middle-income":ab,ti OR "least developed countries":ab,ti OR "underdeveloped*":ab,ti OR "third-world*":ab,ti OR "thirdworld*":ab,ti OR "3rd-world":ab,ti OR "Albania":ab,ti OR "American Samoa":ab,ti OR "Argentina":ab,ti OR "Armenia":ab,ti OR "Azerbaijan":ab,ti OR "Belarus":ab,ti OR "Belize":ab,ti OR "Bosnia and Herzegovina":ab,ti OR "Botswana":ab,ti OR "Brazil":ab,ti OR "Bulgaria":ab,ti OR "China":ab,ti OR "Colombia":ab,ti OR "Costa Rica":ab,ti OR "Cuba":ab,ti OR "Dominica":ab,ti OR "Dominican Republic":ab,ti OR "Ecuador":ab,ti OR "Equatorial Guinea":ab,ti OR "Fiji":ab,ti OR "Gabon":ab,ti OR "Georgia":ab,ti OR "Grenada":ab,ti OR "Guatemala":ab,ti OR "Guyana":ab,ti OR "Indonesia":ab,ti OR "Iran":ab,ti OR "Islamic*":ab,ti OR "Iraq":ab,ti OR "Jamaica":ab,ti OR "Jordan":ab,ti OR "Kazakhstan":ab,ti OR "Kosovo":ab,ti OR "Lebanon":ab,ti OR "Libya":ab,ti OR "Malaysia":ab,ti OR "Maldives":ab,ti OR "Marshall Islands":ab,ti OR "Micronesia":ab,ti OR "Mexico":ab,ti OR "Montenegro":ab,ti OR "Namibia":ab,ti OR "North Macedonia":ab,ti OR "Paraguay":ab,ti OR "Peru":ab,ti OR "Russian*":ab,ti OR "Samoa":ab,ti OR "Serbia":ab,ti OR "South Africa":ab,ti OR "Saint Lucia":ab,ti OR "Saint Vincent*":ab,ti OR "Suriname":ab,ti OR "Thailand":ab,ti OR "Tonga":ab,ti OR "Turkey":ab,ti OR "Turkmenistan":ab,ti OR "Tuvalu":ab,ti OR "Venezuela*":ab,ti |
| #3 | 'economic analysis'/exp OR 'health economics'/mj OR 'economic evaluation'/mj OR 'cost of illness'/mj OR 'health care cost'/mj OR 'economic model'/mj OR 'financial burden'/mj OR 'economic burden'/mj OR 'resource allocation'/mj OR 'out of pocket expenditure'/mj OR 'direct cost'/mj OR "Income*":ab,ti OR "Expenditure*":ab,ti OR "cost-of-illness":ab,ti OR "cost of illness":ab,ti OR "cost of sickness":ab,ti OR "financial burden":ab,ti OR "financial impact":ab,ti OR "economic burden":ab,ti OR "burden of illness":ab,ti OR "out-of-pocket*":ab,ti OR "health expenditure*":ab,ti OR "budget*":ab,ti OR "fee":ab,ti OR "direct cost*":ab,ti OR "cost allocation":ab,ti OR "payment*":ab,ti OR "cost analysis":ab,ti OR "macroeconomic*":ab,ti OR "microeconomic*":ab,ti OR "pricing":ab,ti OR "Cost measure*":ab,ti OR "Illness Burden*":ab,ti OR "sickness cost*":ab,ti OR "disease cost*":ab,ti OR "Illness cost*":ab,ti OR "direct service cost*":ab,ti OR "service cost*":ab,ti OR "hospital charge*":ab,ti OR "hospital cost*":ab,ti OR "hospitalization cost*":ab,ti OR "medical cost*":ab,ti OR "medical care cost*":ab,ti OR "health care cost*":ab,ti OR "health care economics*":ab,ti OR "employer health cost*":ab,ti OR "medical burden":ab,ti OR "spending":ab,ti OR "Markov*":ab,ti OR "Monte Carlo Method":ab,ti OR "Decision Trees":ab,ti |
| #4 | #1 AND #2 AND #3 |
| #5 | #4 AND [embase]/lim NOT ([embase]/lim AND [medline]/lim) |
| **Number of records identified:** 1,036 | |

| **Database: BVS Portal** | |
| --- | --- |
| #1 | mh:"Infecções Respiratórias/EC" OR mh:"Infecções por Coronavirus/EC" OR mh:"Síndrome Respiratória Aguda Grave/EC" OR mh:"Coronavírus da Síndrome Respiratória do Oriente Médio/EC" OR mh:"Influenza Humana/EC" OR mh:"Infecções por Vírus Respiratório Sincicial/EC" OR mh:"Bronquiolite Viral/EC" OR mh:"Pneumonia Viral/EC" OR mh:"Infecções por Adenovirus Humanos/EC" OR mh:"Vírus da Parainfluenza 3 Humana/EC" OR ab:"respiratory tract infection*" OR ab:"Infecciones del Sistema Respiratorio" OR ab:"infecciones por coronavirus" OR ab:"Doença pelo Novo Coronavírus" OR ab:"Coronavirus Disease 2019" OR ab:"Enfermedad por coronavirus" OR ab:"Enfermedad por el nuevo coronavirus" OR ab:"Enfermedad del coronavirus 2019" OR ab:"COVID-19" OR ab:"Severe Acute Respiratory Syndrome" OR ab:"SARS" OR ab:"Middle East Respiratory Syndrome*" OR ab:"Coronavirus del Síndrome Respiratorio do Oriente Medio" OR ab:"MERS" OR ab:"SARS-CoV*" OR ab:"Síndrome Respiratorio Agudo Grave" OR ab:"Gripe Humana" OR ab:"Influenza em Humanos" OR ab:"Respiratory Syncytial Virus Infections" OR ab:"Syncytial Virus*" OR ab:"Infecciones por Virus Sincitial Respiratorio" OR ab:"Vírus Sincicial Respiratório" OR ab:"Bronquiolitis Viral" OR ab:"Viral Bronchiolitis" OR ab:"Viral Pneumonia" OR ab:"Neumonía Viral" OR ab:"Para influenza virus type 3" OR ab:"Para-influenza virus type 3" OR ab:"Human parainfluenza virus 3" OR ab:"para influenza 3" OR ab:"parainfluenza 3*" OR ab:"Parainfluenza Virus 3" OR ab:"Virus de la Parainfluenza 3 humana" OR ab:"Influenza Like*" OR ab:"Influenza-like*" OR ab:"flu like*" OR ab:"flu-like*" |
| #2 | mh:"Países em Desenvolvimento/EC" OR ab:"Developing Countries" OR ab:"Países en Desarrollo" OR ab:"Países subdesenvolvidos" OR ab:"Países do Terceiro Mundo" OR ab:"middle-income" OR ab:"upper-middle-income" OR ab:"least developed countries" OR ab:"third-world*" OR ab:"thirdworld*" OR ab:"3rd-world" OR ab:"Albania" OR ab:"Albânia" OR ab:"Samoa Americana" OR ab:"American Samoa" OR ab:"Argentina" OR ab:"Armênia" OR ab:"Armenia" OR ab:"Azerbaijão" OR ab:"Azerbaijan" OR ab:"Azerbaiyán" OR ab:"Belarus" OR ab:"Belize" OR ab:"Belice" OR ab:"Bósnia e Herzegóvina" OR ab:"Bosnia y Herzegovina" OR ab:"Bosnia and Herzegovina" OR ab:"Botsuana" OR ab:"Botswana" OR ab:"Brazil" OR ab:"Brasil" OR ab:"Bulgária" OR ab:"Bulgaria" OR ab:"China" OR ab:"Colômbia" OR ab:"Colombia" OR ab:"Costa Rica" OR ab:"Cuba" OR ab:"Dominica" OR ab:"República Dominicana" OR ab:"Dominican Republic" OR ab:"Equador" OR ab:"Ecuador" OR ab:"Guiné Equatorial" OR ab:"Guinea Ecuatorial" OR ab:"Equatorial Guinea" OR ab:"Fiji" OR ab:"Gabão" OR ab:"Gabón" OR ab:"Gabon" OR ab:"República da Geórgia" OR ab:"Georgia" OR ab:"Grenada" OR ab:"Guatemala" OR ab:"Guiana" OR ab:"Guyana" OR ab:"Indonesia" OR ab:"Indonésia" OR ab:"Irán" OR ab:"Iran" OR ab:"Irã" OR ab:"República Islâmica*" OR ab:"Iraq" OR ab:"Iraque" OR ab:"Irak" OR ab:"Jamaica" OR ab:"Jordânia" OR ab:"Jordania" OR ab:"Jordan" OR ab:"Cazaquistão" OR ab:"Kazajstán" OR ab:"Kazakhstan" OR ab:"Kosovo" OR ab:"Líbano" OR ab:"Lebanon" OR ab:"Líbia" OR ab:"Libia" OR ab:"Libya" OR ab:"Malásia" OR ab:"Malasia" OR ab:"Malaysia" OR ab:"Maldivas" OR ab:"Maldives" OR ab:"Micronésia" OR ab:"Micronesia" OR ab:"Marshall Islands" OR ab:"México" OR ab:"Mexico" OR ab:"Montenegro" OR ab:"Namíbia" OR ab:"Namibia" OR ab:"Macedônia" OR ab:"Macedonia" OR ab:"Paraguay" OR ab:"Paraguai" OR ab:"Peru" OR ab:"Perú" OR ab:"Samoa" OR ab:"Sérvia" OR ab:"Serbia" OR ab:"África do Sul" OR ab:"Sudáfrica" OR ab:"South Africa" OR ab:"Santa Lucia" OR ab:"Saint Lucia" OR ab:"São Vincente*" OR ab:"San Vincente*" OR ab:"Saint Vincent*" OR ab:"Suriname" OR ab:"Tailândia" OR ab:"Tailandia" OR ab:"Thailand" OR ab:"Tonga" OR ab:"Turquia" OR ab:"Turkey" OR ab:"Turcomenistão" OR ab:"Turkmenistán" OR ab:"Turkmenistan" OR ab:"Tuvalu" OR ab:"Venezuela*" |
| #3 | tw:"economic assessment in health" OR tw:"economic assessment in healthcare" OR tw:"economic evaluation in health*" OR mh:"custos de cuidados de saude" OR tw:"custos de cuidados de saúde" OR tw:"health care costs" OR tw:"costos de la atención en salud" OR tw:"custos de cuidados médicos" OR tw:"custos de tratamento" OR tw:" costos de la atención médica" OR tw:"costos del tratamiento" OR tw:"health cost*" OR tw:"healthcare cost*" OR tw:"health care cost*" OR tw:"medical care cost*" OR tw:"treatment cost*" OR mh:"Financiamento em Saude" OR mh:"Gastos em Saude" OR mh:"Investimentos em Saude" OR mh:"Recursos financeiros em saude" OR mh:"dotacao de recursos para cuidados de saude" OR mh:"cost of illness" OR mh:" Costo de Enfermedad" OR ab:"custo da doença*" OR ab:"fardo da doença" OR ab:"ônus da doença" OR mh:"equidade na alocacao de recursos" OR mh:"Alocacao de recursos" OR mh:"distribuicao de recursos para cuidados de saude" OR mh:"administracao financeira" OR mh:orcamento OR mh:"Custos e analise de custos" OR mh:"Financiamento da assistencia a saude" OR mh:"Sistemas de Saude/OG" OR mh:"Custos e Análise de Custo" OR ti:"Costs and Cost Analysis" OR ti:"Costos y Análisis de Costo" OR mh:"Honorários e Preços" OR ti:"Fees and Charges" OR ti:"Honorarios y Precios" OR mh:Orçamentos OR ti:Orçamento* OR ti:Budget* OR ti:Presupuestos OR ti:"Controle Orçamentário" OR ti:cost OR ti:custo OR ti:costs OR (tw:econom* (ti:custos OR ti:costly OR ti:costing OR ti:custeio OR ti:price OR ti:prices OR ti:pricing OR ti:preço OR ti:preços OR ti:expenditure OR ti:expenditures OR ti:expense* OR ti:expenses OR ti:despesa* OR ti:financ*)) OR ti:custos OR ti:costly OR ti:costing OR ti:custeio OR ti:price OR ti:prices OR ti:pricing OR ti:preço OR ti:preços OR ti:expenditure OR ti:expenditures OR ti:expense* OR ti:expenses OR ti:despesa* OR ti:gastos OR ti:financ* OR ti:"value for money" OR ti:"monetary value" OR ti:"valor monetário" OR mh:"Modelos Econômicos" OR ti:"Modelos Econômicos" OR ti:"Economic Models" OR ti:"Economic Model" OR mh:"Cadeias de Markov" OR ti:"Cadeias de Markov" OR ti:"Markov Chains" OR ti:"Cadenas de Markov" OR ti:markov OR mh:"Método de Monte Carlo" OR ti:"Método de Monte Carlo" OR ti:"Carlo Method" OR ti:"Método de Montecarlo" OR ti:"monte carlo" OR ti:"decision tree" OR ti:"decision treeboost" OR ti:"decision trees" |
| #4 | #1 AND #2 AND #3 |
| **Number of records identified:** 305 | |

| **Database: CINAHL via EBSCO** | |
| --- | --- |
| #1 | (MM "Respiratory Tract Infections/EC") OR (MM "Coronavirus Infections/EC") OR (MM "COVID-19/EC") OR (MM "Severe Acute Respiratory Syndrome/EC") OR (MM "Middle East Respiratory Syndrome/EC") OR (MM "Influenza, Human/EC") OR (MM "Respiratory Syncytial Virus Infections/EC") OR (MM "Bronchiolitis/EC") OR (MM "Pneumonia, Viral/EC") OR (MM "SARS Virus") OR (MM "Middle East Respiratory Syndrome Coronavirus") OR (AB "viral respiratory infection") OR (AB "viral respiratory diseases") OR (AB "acute respiratory tract infection") OR (AB "airway infection") OR (AB "pulmonary tract infection") OR (AB "coronavirus") OR (AB "SARS-CoV") OR (AB "SARS-CoV-2") OR (AB "COVID-19") OR (AB "Coronavirus Disease 2019") OR (AB "Coronavirus Pandemic") OR (AB "severe acute respiratory syndrome") OR (AB "SARS") OR (AB "acute respiratory disease") OR (AB "MERS") OR (AB "Syncytial virus") OR (TI "Syncytial virus") OR (AB "viral pneumonia") OR (AB "parainfluenza virus type 3") OR (AB "para influenza virus type 3") OR (AB "para-influenza virus type 3") OR (AB "bronchiolitis") OR (AB "influenza") OR (TI "influenza like illness") OR (AB "influenza like illness") OR (AB "influenza-like illness") OR (TI "influenza-like illness") OR (AB "influenza like symptom") OR (AB "influenza like syndrome") OR (AB "flu like illness") OR (AB "flu like symptom") OR (AB "acute influenzal pneumonia") |
| #2 | (MH "Developing Countries") OR (AB "middle-income") OR (AB "upper-middle-income") OR (AB "least developed countries") OR (AB "under developed nations") OR (AB "underdeveloped") OR (AB "third-world") OR (AB "thirdworld") OR (AB "3rd-world") OR (AB "Albania") OR (AB "American Samoa") OR (AB "Argentina") OR (AB "Armenia") OR (AB "Azerbaijan") OR (AB "Belarus") OR (AB "Belize") OR (AB "Bosnia and Herzegovina") OR (AB "Botswana") OR (AB "Brazil") OR (AB "Bulgaria") OR (AB "China") OR (AB "Colombia") OR (AB "Costa Rica") OR (AB "Cuba") OR (AB "Dominica") OR (AB "Dominican Republic") OR (AB "Ecuador") OR (AB "Equatorial Guinea") OR (AB "Fiji") OR (AB "Gabon") OR (AB "Georgia") OR (AB "Grenada") OR (AB "Guatemala") OR (AB "Guyana") OR (AB "Indonesia") OR (AB "Iran") OR (AB "Islamic") OR (AB "Iraq") OR (AB "Jamaica") OR (AB "Jordan") OR (AB "Kazakhstan") OR (AB "Kosovo") OR (AB "Lebanon") OR (AB "Libya") OR (AB "Malaysia") OR (AB "Maldives") OR (AB "Marshall Islands") OR (AB "Micronesia") OR (AB "Mexico") OR (AB "Montenegro") OR (AB "Namibia") OR (AB "North Macedonia") OR (AB "Paraguay") OR (AB "Peru") OR (AB "Russian") OR (AB "Samoa") OR (AB "Serbia") OR (AB "South Africa") OR (AB "Saint Lucia") OR (AB "Saint Vincent") OR (AB "Suriname") OR (AB "Thailand") OR (AB "Tonga") OR (AB "Turkey") OR (AB "Turkmenistan") OR (AB "Tuvalu") OR (AB "Venezuela") |
| #3 | (TI "budget") OR (AB "budget") OR (AB "burden of illness") OR (AB "Carlo Method") OR (AB "cost allocation") OR (TI "cost analysis") OR (AB "cost analysis") OR (AB "Cost measures") OR (TI "cost of illness") OR (AB "cost of illness") OR (AB "cost of sickness") OR (TI "cost-of-illness") OR (AB "cost-of-illness") OR (TI "Cost Analysis") OR (AB "Cost Analysis") OR (AB "decision tree") OR (AB "decision treeboost") OR (AB "decision trees") OR (TI "direct cost") OR (AB "direct cost") OR (AB "direct service cost") OR (AB "disease cost") OR (TI "economic analysis") OR (AB "economic analysis") OR (TI "economic assessment") OR (AB "economic assessment") OR (TI "economic burden") OR (AB "economic burden") OR (TI "economic evaluation") OR (AB "economic evaluation") OR (AB "Economic Model") OR (AB "Economic Models") OR (AB "employer health cost") OR (TI "Expenditure") OR (AB "Expenditure") OR (AB "fee") OR (AB "financial burden") OR (TI "financial impact") OR (AB "financial impact") OR (AB "health care cost") OR (AB "health care costs") OR (AB "health care economics") OR (AB "health costs") OR (AB "health economics") OR (AB "health expenditure") OR (AB "healthcare cost") OR (AB "hospital charge") OR (AB "hospital cost") OR (TI "hospitalization cost") OR (AB "hospitalization cost") OR (TI "Illness Burden") OR (AB "Illness Burden") OR (AB "Illness cost") OR (AB "Income") OR (AB "Markov Chains") OR (AB "medical burden") OR (AB "medical care costs") OR (AB "medical care cost") OR (AB "medical costs") OR (AB "Monte Carlo Method") OR (AB "out of pocket") OR (AB "out-of-pocket") OR (AB "payment") OR (AB "pricing") OR (AB "resource allocation") OR (AB "service costs") OR (AB "sickness costs") OR (AB "spending") |
| #4 | #1 AND #2 AND #3 |
| **Number of records identified:** 257 | |

| **Database: CRD Library** | |
| --- | --- |
| #1 | "Respiratory Tract Infections" OR "Coronavirus Infections" OR "COVID-19" OR "Severe Acute Respiratory Syndrome" OR "Middle East Respiratory Syndrome" OR "Respiratory Syncytial Virus Infections" OR "Bronchiolitis" OR "Viral Pneumonia" OR "Middle East Respiratory Syndrome Coronavirus" OR "viral respiratory infection" OR "viral respiratory diseases" OR "acute respiratory tract infection" OR "airway infection" OR "pulmonary tract infection" OR "coronavirus" OR "SARS-CoV" OR "SARS-CoV-2" OR "COVID-19" OR "Coronavirus Disease 2019" OR "Coronavirus Pandemic" OR "SARS" OR "acute respiratory disease" OR "MERS" OR "Syncytial virus" OR "parainfluenza virus type 3" OR "para influenza virus type 3" OR "para-influenza virus type 3" OR "bronchiolitis" OR "influenza" OR "influenza like illness" OR "influenza-like illness" OR "influenza like symptom" OR "influenza like syndrome" OR "flu like illness" OR "flu like symptom" OR "acute influenzal pneumonia" |
| #2 | "budget" OR "burden of illness" OR "Carlo Method" OR "cost allocation" OR "cost analysis" OR "Cost measures" OR "cost of illness" OR "cost of sickness" OR "cost-of-illness" OR "Cost Analysis" OR "decision tree" OR "decision treeboost" OR "decision trees" OR "direct cost" OR "direct service cost" OR "disease cost" OR "economic analysis" OR "economic assessment" OR "economic burden" OR "economic evaluation" OR "Economic Model" OR "Economic Models" OR "employer health cost" OR "Expenditure" OR "fee" OR "financial burden" OR "financial impact" OR "health care cost" OR "health care costs" OR "health care economics" OR "health costs" OR "health economics" OR "health expenditure" OR "healthcare cost" OR "hospital charge" OR "hospital cost" OR "hospitalization cost" OR "Illness Burden" OR "Illness cost" OR "Markov Chains" OR "medical burden" OR "medical care costs" OR "medical care cost" OR "medical costs" OR "Monte Carlo Method" OR "out of pocket" OR "out-of-pocket" OR "payment" OR "pricing" OR "resource allocation" OR "service costs" OR "sickness costs" OR "spending" |
| #3 | #1 AND #2 IN DARE, NHSEED, HTA |
| **Number of records identified:** 363 | |

| **Database: MedRxiv*** | |
| --- | --- |
| 1^st^ | *For abstract or title* ("Coronavirus" OR "COVID-19" OR "SARS-CoV-2" OR "COVID-19" OR "Coronavirus Disease 2019" OR "Coronavirus Pandemic") AND (Limit Subject Area: Health Economics) |
| **Number of records identified:** 138 | |
| 2^nd^ | *For abstract or title* ("Severe Acute Respiratory Syndrome" OR "SARS" OR "viral respiratory diseases" OR "acute respiratory disease") AND (Limit Subject Area: Health Economics) |
| **Number of records identified:** 78 | |
| 3^rd^ | *For abstract or title* ("Middle East Respiratory Syndrome" OR "MERS" OR "Middle East Respiratory Syndrome Coronavirus") AND (Limit Subject Area: Health Economics) |
| **Number of records identified:** 32 | |
| 4^th^ | *For abstract or title* ("Respiratory Syncytial Virus Infections" OR "Syncytial virus" OR "Bronchiolitis" OR "Viral Pneumonia") AND (Limit Subject Area: Health Economics) |
| **Number of records identified:** 16 | |
| 5^th^ | *For abstract or title* ("parainfluenza virus type 3" OR "para influenza virus type 3" OR "para-influenza virus type 3") AND (Limit Subject Area: Health Economics) |
| **Number of records identified:** 93 | |
| 6^th^ | *For abstract or title* ("influenza like" OR "influenza-like illness" OR "flu like illness" OR "flu like symptom") AND (Limit Subject Area: Health Economics) |
| **Number of records identified:** 9 | |
| *Due to limitations of MedRxiv platform, we performed six independent search strategies. | |

| **Database: Research Square** |
| --- |
| *Filter Abstract:* ("budget" OR "burden of illness" OR "Carlo Method" OR "cost allocation" OR "cost analysis" OR "Cost measures" OR "cost of illness" OR "cost of sickness" OR "cost-of-illness" OR "Cost Analysis" OR "decision tree" OR "decision treeboost" OR "decision trees" OR "direct cost" OR "direct service cost" OR "disease cost" OR "economic analysis" OR "economic assessment" OR "economic burden" OR "economic evaluation" OR "Economic Model" OR "Economic Models" OR "employer health cost" OR "Expenditure" OR "fee" OR "financial burden" OR "financial impact" OR "health care cost" OR "health care costs" OR "health care economics" OR "health costs" OR "health economics" OR "health expenditure" OR "healthcare cost" OR "hospital charge" OR "hospital cost" OR "hospitalization cost" OR "Illness Burden" OR "Illness cost" OR "Markov Chains" OR "medical burden" OR "medical care costs" OR "medical care cost" OR "medical costs" OR "Monte Carlo Method" OR "out of pocket" OR "out-of-pocket" OR "payment" OR "pricing" OR "resource allocation" OR "service costs" OR "sickness costs" OR "spending") AND *Filter Title:* ("Respiratory Tract Infections" OR "Coronavirus Infections" OR "COVID-19" OR "Severe Acute Respiratory Syndrome" OR "Middle East Respiratory Syndrome" OR "Respiratory Syncytial Virus Infections" OR "Bronchiolitis" OR "Viral Pneumonia" OR "Middle East Respiratory Syndrome Coronavirus" OR "viral respiratory infection" OR "viral respiratory diseases" OR "acute respiratory tract infection" OR "airway infection" OR "pulmonary tract infection" OR "coronavirus" OR "SARS-CoV" OR "SARS-CoV-2" OR "COVID-19" OR "Coronavirus Disease 2019" OR "Coronavirus Pandemic" OR "SARS" OR "acute respiratory disease" OR "MERS" OR "Syncytial virus" OR "parainfluenza virus type 3" OR "para influenza virus type 3" OR "para-influenza virus type 3" OR "bronchiolitis" OR "influenza" OR "influenza like illness" OR "influenza-like illness" OR "influenza like symptom" OR "influenza like syndrome" OR "flu like illness" OR "flu like symptom" OR "acute influenzal pneumonia") |
| **Number of records identified:** 106 |
