# Supplementary material for: Hospitalization costs of coronaviruses diseases in upper-middle-income countries: A systematic review
Source: PLoS One. 2022 Mar 11;17(3):e0265003. doi: 10.1371/journal.pone.0265003 (PMC8916657; doi:10.1371/journal.pone.0265003)
Supplement: S2 Table — (DOC) [file pone.0265003.s004.doc]

# S2 Table. Characteristics of excluded studies

| **Study ID [ordered by study ID]** | **Reason for exclusion** |
| --- | --- |
| Beutels et al. (2009) [1] | Study reporting only intangible costs. |
| Qiu et al. (2018) [2] | Not an economic analysis. |
| Andrade et al. (2020) [3] | Study reporting only indirect costs. |
| Darab et al. (2020) [4] | Preprint publication of an included study. |
| Jin et al. (2020) [5] | Preprint publication of an included study. |
| Kirigia and Muthuri (2020) [6] | Study reporting only intangible costs. |
| Kolbin et al. (2020) [7] | Abstract publication of an included “study awaiting classification”. |
| Squire et al. (2020) [8] | Intervention costs only. |
