# Supplementary material for: Hospitalization costs of coronaviruses diseases in upper-middle-income countries: A systematic review
Source: PLoS One. 2022 Mar 11;17(3):e0265003. doi: 10.1371/journal.pone.0265003 (PMC8916657; doi:10.1371/journal.pone.0265003)
Supplement: S3 Table — (DOC) [file pone.0265003.s005.doc]

# S3 Table. Characteristics of studies awaiting classification

| **Study ID [ordered by study ID]** | **Criteria for classification** |
| --- | --- |
| Xiao et al. (2004) [9] | Abstract without access to full text. |
| Kolbin et al. (2020) [10] | Study in non-Roman language. |
