# Supplementary material for: Hospitalization costs of coronaviruses diseases in upper-middle-income countries: A systematic review
Source: PLoS One. 2022 Mar 11;17(3):e0265003. doi: 10.1371/journal.pone.0265003 (PMC8916657; doi:10.1371/journal.pone.0265003)
Supplement: S4 Table — (DOC) [file pone.0265003.s006.doc]

# S4 Table. Medical cost categories definitions reported in the included studies

| **Study ID** | **Medical cost category: definition/component*** |
| --- | --- |
| Darab et al. (2021) [11] | Physician visit cost: “in Iran’s health system cost of the visit includes the fee that is paid for each time a physician a patient at their bedside to examine or prescribe them”. |
|  | Other services: “including monitoring in non-intensive care units, compilation and prescription of therapeutic diets, cannula implantation for venous-venous-hemodialysis, blood and blood products transfusion, tracheal intubation, level-one critical care or triage, blood oximetry or continuous pulse oximetry, temporary catheter insertion into the bladder, and cold room”. |
| Jin et al. (2021) [12] | Follow-up appointment: “Two follow-up appointments are recommended: one at week two and one at week four, after hospital discharge”. |
|  | Inpatient care: “Hospital bed days, nursing, blood gas analyses and laboratory tests”. |
|  | Medicines: “Anti-infective medicines and nutrition support”. |
| *As presented by the authors. | |
