# Supplementary material for: Hospitalization costs of coronaviruses diseases in upper-middle-income countries: A systematic review
Source: PLoS One. 2022 Mar 11;17(3):e0265003. doi: 10.1371/journal.pone.0265003 (PMC8916657; doi:10.1371/journal.pone.0265003)
Supplement: S5 Table — (DOC) [file pone.0265003.s007.doc]

# S5 Table. Report assessment tool

*Adapted from Oliveira, Itria and Lima (2019)* [13]*.*

| **Section** | **Q#** | **Item** |
| --- | --- | --- |
| Drawing the study | Q01 | Research is adequate? |
|  | Q02 | The epidemiological source is stated? |
|  | Q03 | The study is identified as an economic evaluation? |
|  | Q04 | Provide a structured summary? |
|  | Q05 | Describe characteristics of population? |
|  | Q06 | The authors reported time horizon? |
|  | Q07 | The authors reported study perspective? |
|  | Q08 | The form of economic evaluation used is stated? |
|  | Q09 | The study was approved by an institution authorized in ethics in research? |
|  | Q10 | The authors reported any conflicts of interest? |
|  | Q11 | The authors reported if the study was funded? |
| Data Collection | Q12 | The source(s) of costs estimates used are stated? |
|  | Q13 | The costs were clearly described? |
|  | Q14 | The valuation method is stated? |
|  | Q15 | Type of cost is stated? |
|  | Q16 | The authors reported currency, price date, and conversion? |
|  | Q17 | The analytical model used is stated? |
|  | Q18 | There is justification for methods and assumptions for extrapolating results? |
|  | Q19 | The measurement of costs is adequate? |
| Analysis and Interpretation  of the Results | Q20 | The information was based on evidence of quality? |
|  | Q21 | The authors characterized uncertainty? |
|  | Q22 | The authors reported heterogeneity? |
|  | Q23 | Outcome measures in health were clearly described and relevant to the study question? |
|  | Q24 | The results were presented as ratio between health costs and outcomes? |
|  | Q25 | The approach to sensitivity analysis is given? |
|  | Q26 | The results section reported relevant aspects? |
|  | Q27 | The variation of costs over time is justified? |
|  | Q28 | Conclusions follow from the data reported? |
|  | Q29 | Conclusions are accompanied by the appropriate caveats? |
