# Supplementary material for: Hospitalization costs of coronaviruses diseases in upper-middle-income countries: A systematic review
Source: PLoS One. 2022 Mar 11;17(3):e0265003. doi: 10.1371/journal.pone.0265003 (PMC8916657; doi:10.1371/journal.pone.0265003)
Supplement: S1 Appendix — (DOC) [file pone.0265003.s008.doc]

# S1 Appendix. Supporting information references

1. Beutels P, Jia N, Zhou Q-Y, Smith R, Cao W-C, de Vlas SJ. The economic impact of SARS in Beijing, China. Trop. Med. Int. Heal. 2009 Nov;14:85–91. doi: 10.1111/j.1365-3156.2008.02210.x.

2. Qiu W, Chu C, Mao A, Wu J. The Impacts on Health, Society, and Economy of SARS and H7N9 Outbreaks in China: A Case Comparison Study. J. Environ. Public Health. 2018 Jun 28;2018:1–7. doi: 10.1155/2018/2710185.

3. Andrade R, Sansone D, Farah D, Garcia R, Tannus G, Fonseca M. PMH16 Productivity, Depression, and Measures to Reduce Social IMPACT: The Economic IMPACT of COVID-19 in Brazil. Value Heal. 2020 Dec;23:S587. doi: 10.1016/j.jval.2020.08.1094.

4. Darab MG, Keshavarz K, Sadeghi E, Shahmohamadi J, Kasovi Z. The Economic Burden of Coronavirus Disease 2019 (COVID-19): Evidence from Iran. 2020; PREPRINT (Version 1) available at Research Square. doi: 10.21203/rs.3.rs-80962/v1.

5. Jin H, Wang H, Li X, Zheng W, Ye S, Zhang S, et al. Estimating the Cost-of-Illness Associated with the COVID-19 Outbreak in China from January to March 2020. medRxiv. 2020. doi: 10.1101/2020.05.15.20102863.

6. Kirigia JM, Muthuri RNDK. The fiscal value of human lives lost from coronavirus disease (COVID-19) in China. BMC Res. Notes. 2020 Dec 1;13(1):198. doi: 10.1186/s13104-020-05044-y.

7. Kolbin A, Gomon J, Balykina Y, Belousov D, Ivanov I. PIN65 Socio-Economic Burden of COVID-19 in Russian Federation. Value Heal. 2020 Dec;23:S556. doi: 10.1016/j.jval.2020.08.906.

8. Squire MM, Munsamy M, Lin G, Telukdarie A, Igusa T. Modeling Hospital Energy and Economic Costs for COVID-19 Infection Control Interventions. medRxiv. 2020. doi: 10.1101/2020.08.21.20178855.

9. Xiao F, Chen B, Wu Y, Wang Y, Han D, Beijing (Provisional) Commanding Center For SARS Treatment and Cure Scientific Research Group. [Analysis on the cost and its related factors of clinically confirmeds severe acute respiratory syndrome cases in Beijing]. Zhonghua Liu Xing Bing Xue Za Zhi. 2004 Apr;25(4):312–6. PMID: 15231198.

10. Kolbin AS, Belousov DY, Gomon YM, Balykina YE, Ivanov IG. Socio-economic burden of COVID-19 in the Russian Federation. Kachestvennaya Klin. Prakt. 2020 May 26;(1):35–44. doi: 10.37489/2588-0519-2020-1-35-44.

11. Darab MG, Keshavarz K, Sadeghi E, Shahmohamadi J, Kavosi Z. The economic burden of coronavirus disease 2019 (COVID-19): evidence from Iran. BMC Health Serv. Res. 2021 Dec 11;21(1):132. doi: 10.1186/s12913-021-06126-8.

12. Jin H, Wang H, Li X, Zheng W, Ye S, Zhang S, et al. Economic burden of COVID-19, China, January–March, 2020: a cost-of-illness study. Bull. World Health Organ. 2021 Feb 1;99(2):112–124. doi: 10.2471/BLT.20.267112.

13. Oliveira LN da S, Itria A, Lima EC. Cost of illness and program of dengue: A systematic review. PLoS One. 2019;14(2):e0211401. doi: 10.1371/journal.pone.0211401

14. Gedik H. The cost analysis of inpatients with COVID-19. Acta Medica Mediterr. 2020;36(6):3289. doi: 10.19193/0393-6384_2020_6_520.

15. Liang X, Xiao L, Yang X-L, Zhong X, Zhang P, Tang X, et al. Economic Burden of Public Health Care Was Higher Than That of Hospitalization and Treatment Associated With COVID-19 in China. 2020; PREPRINT (Version 1) available at Research Square. doi: 10.21203/rs.3.rs-79298/v1.

16. Miethke-Morais A, Cassenote A, Piva H, Tokunaga E, Cobello V, Rodrigues Gonçalves FA, et al. Unraveling COVID-19-related hospital costs: The impact of clinical and demographic conditions. medRxiv. 2020 Jan 1;2020.12.24.20248633. doi: 10.1101/2020.12.24.20248633.

17. McGuinness LA, Higgins JPT. Risk‐of‐bias VISualization (robvis): An R package and Shiny web app for visualizing risk‐of‐bias assessments. Res. Synth. Methods. 2021 Jan 6;12(1):55–61. doi: 10.1002/jrsm.1411.
